# Intranasal Bacterial Therapeutics Reduce Colonization by the Respiratory Pathogen Mannheimia haemolytica in Dairy Calves

**DOI:** 10.1128/mSystems.00629-19

**Published:** 2020-03-03

**Authors:** Samat Amat, Trevor W. Alexander, Devin B. Holman, Timothy Schwinghamer, Edouard Timsit

**Affiliations:** a Agriculture and Agri-Food Canada, Lethbridge Research and Development Centre, Lethbridge, Alberta, Canada; b Faculty of Veterinary Medicine, University of Calgary, Calgary, Alberta, Canada; c Lacombe Research and Development Centre, Agriculture and Agri-Food Canada, Lacombe, Alberta, Canada; d Simpson Ranch Chair in Beef Cattle Health and Wellness, University of Calgary, Calgary, Alberta, Canada; e CEVA Santé Animale, Libourne, France; Institute for Systems Biology

**Keywords:** bacterial therapeutics, bovine respiratory disease, *Lactobacillus* spp., *Mannheimia haemolytica*, respiratory microbiota, dairy calves

## Abstract

Six *Lactobacillus* strains originating from the nasopharyngeal microbiota of cattle were previously characterized *in vitro* and identified as candidate bacterial therapeutics (BTs) for mitigating the bovine respiratory pathogen Mannheimia haemolytica. In the present study, these BT strains were evaluated for their potential to (i) reduce nasal colonization by *M. haemolytica,* (ii) modulate the nasal microbiota, and (iii) stimulate an immune response in calves experimentally challenged with *M. haemolytica.* Twenty-four Holstein bull calves (1 to 3 weeks old) received either an intranasal BT cocktail containing 6 *Lactobacillus* strains (3 × 10^9^ CFU per strain; BT + Mh group) 24 h prior to intranasal *M. haemolytica* challenge (3 × 10^8^ CFU) or no BTs prior to challenge (Mh, control group). Nasal swab, blood, and transtracheal aspiration samples were collected over the course of 16 days after BT inoculation. Counts of *M. haemolytica* were determined by culturing, and the nasal and tracheal microbiotas were evaluated using 16S rRNA gene sequencing. Serum cytokines (interleukin-6 [IL-6], IL-8, and IL-10) were quantified by enzyme-linked immunosorbent assay (ELISA). Administration of BT reduced nasal colonization by *M. haemolytica* (*P = *0.02), modified the composition and diversity of the nasal microbiota, and altered interbacterial relationships among the 10 most relatively abundant genera. The BT + Mh calves also had a lower relative abundance of *Mannheimia* in the trachea (*P < *0.01) but similar cytokine levels as Mh calves. This study demonstrated that intranasal BTs developed from the bovine nasopharyngeal *Lactobacillus* spp. were effective in reducing nasal colonization by *M. haemolytica* in dairy calves.

**IMPORTANCE** Bovine respiratory disease (BRD) is one of the significant challenges for the modern dairy industry in North America, accounting for 23 to 47% of the total mortality among pre- and postweaned dairy heifers. Mass medication with antibiotics is a common practice to control BRD in dairy cattle. However, the emergence of multidrug-resistant BRD pathogens highlights the importance of developing alternatives to antibiotics for BRD mitigation. Using a targeted approach, we recently identified 6 *Lactobacillus* strains originating from the bovine respiratory microbiota as candidates to be used as bacterial therapeutics (BTs) for the mitigation of the BRD pathogen Mannheimia haemolytica. Here, we demonstrated that intranasal inoculation of the BT strains reduced nasal colonization by *M. haemolytica* in dairy calves experimentally challenged with this pathogen. This study, for the first time, shows the potential use of intranasal BTs as an alternative to mitigate BRD pathogens in cattle.

## Abstract (precis)

Bovine respiratory disease (BRD) is one of the significant challenges for the modern dairy industry in North America, accounting for 23 to 47% of the total mortality among pre- and postweaned dairy heifers. Mass medication with antibiotics is a common practice to control BRD in dairy cattle. However, the emergence of multidrug-resistant BRD pathogens highlights the importance of developing alternatives to antibiotics for BRD mitigation. Using a targeted approach, we recently identified 6 *Lactobacillus* strains originating from the bovine respiratory microbiota as candidates to be used as bacterial therapeutics (BTs) for the mitigation of the BRD pathogen Mannheimia haemolytica. Here, we demonstrated that intranasal inoculation of the BT strains reduced nasal colonization by *M. haemolytica* in dairy calves experimentally challenged with this pathogen. This study, for the first time, shows the potential use of intranasal BTs as an alternative to mitigate BRD pathogens in cattle.

## INTRODUCTION

Bovine respiratory disease (BRD), also known as enzootic calf pneumonia, is one of the most common diseases in dairy heifers and poses a significant welfare and economic burden on the dairy industry in North America (1, 2) and Europe (3). This disease is caused by many contributing factors, including bacteria, viruses, and management and environmental stressors (4). Among bacterial pathogens involved in BRD, Mannheimia haemolytica, Pasteurella multocida, Histophilus somni, and Mycoplasma bovis are considered the most important (5). These pathogens can also colonize the upper respiratory tract (URT) of healthy calves without causing disease (6). However, when respiratory defenses are compromised due to stress or viral infection, pathogens can proliferate in the URT and translocate into the lung, causing bronchopneumonia (7). Hence, limiting the proliferation of these pathogens in the URT may prevent BRD in dairy calves.

Currently, control of bacterial pathogen proliferation and lung infection is largely reliant on the use of antibiotics, which are often given as group medication as early as 10 days of age (4, 8). Unfortunately, this approach is not always effective in preventing BRD (9) and can be seen as an irrational use of antibiotics leading to the development of antibiotic resistance in the digestive and respiratory microbiota (10). This has been shown by the recent characterization of *M. haemolytica* and P. multocida displaying multidrug resistance (11, 12), which in some instances was linked to resistance genes contained within integrative conjugative elements (13, 14). Discovery and development of alternatives to antibiotics to prevent BRD in dairy calves are therefore needed.

Increasing evidence indicates that commensal bacteria in the bovine nasopharynx may prevent the colonization and proliferation of bacterial pathogens through a variety of mechanisms, including direct antagonism (i.e., antimicrobial properties), competition for nutrients and adhesion sites, and host immunomodulatory effects (15–18). Among commensal bacteria, lactic acid-producing bacteria (LAB), and more specifically *Lactobacillus* spp., may be important in providing colonization resistance against bacterial respiratory pathogens (19–21). In a previous study, we used a targeted approach to characterize bacterial therapeutic (BT) candidates originating from the bovine nasopharynx (22). In total, six *Lactobacillus* strains from an initial group of 178 LAB isolates were identified to have the greatest potential as BTs based on their ability to inhibit the growth of *M. haemolytica in vitro*, adhere to and exclude *M. haemolytica* from bovine turbinate cells, and modulate expression of genes related to bacterial infection in turbinate cells.

**FIG 1:**
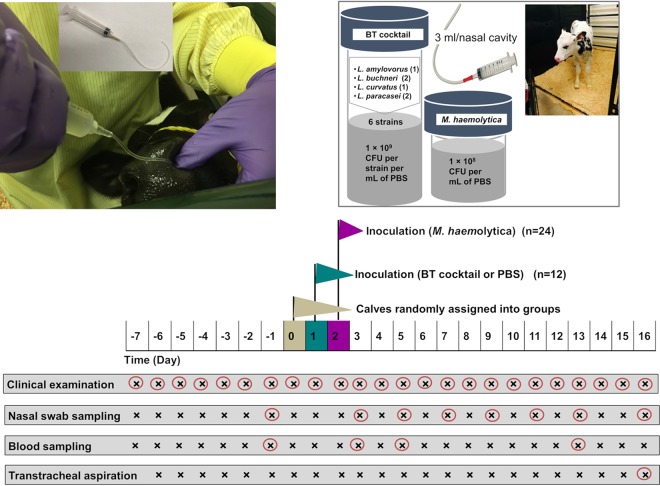
Study design and sampling regimen. The “×” is circled in red if the clinical examination or specific sampling was conducted on that day.

In the current study, we further characterized these BT strains by testing their effectiveness *in vivo* in reducing nasal colonization by *M. haemolytica*, modulating the nasal microbiota, and stimulating an immune response in dairy calves challenged with *M. haemolytica*. Nasal colonization by BT strains and *M. haemolytica*, nasal and tracheal microbiota composition, and serum cytokine concentrations were compared between two groups of dairy calves that were either administered BTs 24 h prior to *M. haemolytica* challenge (BT + Mh) or not administered BTs prior to challenge (Mh) (Fig. 1). As part of a safety assessment of the BTs, gross pathological examination of the lungs was also conducted at the end of the study period.

## RESULTS

### Animal health.

None of the calves displayed abnormal clinical signs (e.g., lethargy, diarrhea, cough, nasal discharge, and increased respiratory rate) throughout the study period. Mean rectal temperatures and respiratory rates were within normal ranges (Table 1; see also Table S1 in the supplemental material) and did not differ between BT + Mh and Mh groups (*P* > 0.05) (Table S1). Gross examination of the lungs at day 16 revealed that 10 calves had lung consolidation. However, percentages of consolidation were low (median = 4.6%; minimum = 0.8%; maximum = 22.6%) and did not differ between groups (*P* > 0.05) (Fig. S1).

**TABLE 1:** Mean rectal temperature and respiratory rate in dairy calves that received intranasal inoculation of either bacterial therapeutics and *M. haemolytica* or only *M. haemolytica*

Day of expt	Rectal temp (°C) (mean ± SD)		Respiratory rate/min (mean ± SEM)	
	Mh	BT + Mh	Mh	BT + Mh
−1	38.4 ± 0.40	38.3 ± 0.23	26 ± 1.5	24 ± 0.9
3	38.5 ± 0.27	38.3 ± 0.57	25 ± 1.0	23 ± 1.0
5	38.6 ± 0.19	38.7 ± 0.44	28 ± 0.9	26 ± 1.1
7	38.6 ± 0.38	38.5 ± 0.22	28 ± 0.9	29 ± 1.4
9	38.7 ± 0.54	38.5 ± 0.23	31 ± 1.6	32 ± 2.6
11	38.5 ± 0.54	38.4 ± 0.21	30 ± 1.1	28 ± 0.9
13	38.7 ± 0.42	38.7 ± 0.41	30 ± 1.1	31 ± 1.3
16	38.6 ± 0.48	38.5 ± 0.30	33 ± 2.5	36 ± 2.7

10.1128/mSystems.00629-19.1 FIG S1 Lung gross pathology of the dairy calves (*n* = 12) that received an intranasal inoculation of either *M. haemolytica* and PBS (Mh) or bacterial therapeutics and *M. haemolytica* (BT + Mh). (A) Proportion of animals that had lung lesions. (B) The severity of the lung lesions measured by lung lesion scoring. (C) Example of a healthy lung from a probiotic animal (calf no. 07-R2) (right) and a lung from a control animal (calf no. 03-R2) displaying lesions (left, dark color). Download FIG S1, TIF file, 2.2 MB. © Crown copyright 2020. 2020 Crown This content is distributed under the terms of the Creative Commons Attribution 4.0 International license.

10.1128/mSystems.00629-19.4 TABLE S1 Comparison of rectal temperature and respiratory rate between dairy calves receiving intranasal inoculation of either bacterial therapeutics and *M. haemolytica* (BT + Mh) or only *M. haemolytica* (Mh). Download Table S1, PDF file, 0.1 MB. © Crown copyright 2020. 2020 Crown This content is distributed under the terms of the Creative Commons Attribution 4.0 International license.

### Isolation and enumeration of *M. haemolytica* from NS.

**FIG 2:**
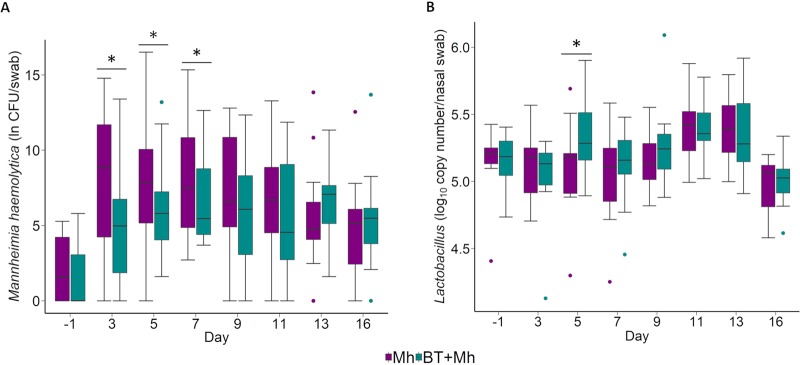
Bacterial counts of *M. haemolytica* determined by culturing (A) and estimated quantity of total *Lactobacillus* spp. determined by qPCR of *Lactobacillus* 16S rRNA gene copies (B) from nasal swabs of calves that received an intranasal inoculation of either bacterial therapeutics and *M. haemolytica* (BT + Mh; *n* = 12) or only *M. haemolytica* (Mh; *n* = 12). Each box indicates the interquartile range (IQR) (middle 50% of the data), the middle line represents the median value, and the whiskers represent 1.5 times the IQR. Colored dots indicate outliers. *, significant difference between treatments (*P* < 0.05).

*M. haemolytica*-specific PCR analysis confirmed that >98% of isolates that were subcultured were *M. haemolytica*. Bacterial counts obtained from culturing the nasal swabs (NS) revealed that BT administration was significantly associated with a 3.3-natural-log-CFU reduction in *M. haemolytica* per NS (*P* = 0.02; Table 2). As shown in Fig. 2A, there was a significant increase in *M. haemolytica* counts in both groups on day 3 (i.e., 24 h after *M. haemolytica* inoculation) over day −1 (*P < *0.05). However, *M. haemolytica* counts remained significantly lower in BT + Mh calves on days 3, 5, and 7 than in Mh calves (*P < *0.05). Effects of time (*P* < 0.01), replicate (*P* = 0.01), and interactions between time and treatment (*P* = 0.02) on *M. haemolytica* counts were also detected (Table 2).

**TABLE 2:** Comparison of *M. haemolytica* counts determined by nasal swab culturing between dairy calves that received intranasal inoculation of either bacterial therapeutics and *M. haemolytica* or only *M. haemolytica*

Independent variable and level^*a*^	Estimated mean bacterial count (natural log CFU/nasal swab) (SE)	*P* value
Treatment		
Mh (*n* = 12)	Reference	
BT + Mh (*n* = 12)	−3.31 (1.36)	0.024
Day of sampling	−0.25 (0.08)	0.003
Treatment × time interaction	0.26 (0.11)	0.025
Replicate no.	2.38 (0.84)	0.010

a Intercept = 7.93 ± 1.049 (*P *< 0.001). Random effect = calf number (*n* = 24).

### PFGE typing of *M. haemolytica* isolates.

The pulsed-field gel electrophoresis (PFGE) typing of PCR-confirmed *M. haemolytica* isolates showed that the inoculated Guelph 1 strain, as indicated by pulsotype, was not isolated from calves prior to its administration on day 3 (Fig. S2). The Guelph 1 strain was the most dominant *M. haemolytica* strain isolated from calves after day 3, indicating a high rate of colonization of calves with this strain. However, six additional pulsotypes were observed, showing that some calves were colonized with different strains of *M. haemolytica*, but these strains were less frequently observed.

10.1128/mSystems.00629-19.2 FIG S2 Phylogenetic tree of pulsed-field gel electrophoresis profiles of *M. haemolytica* isolates. Not all confirmed *M. haemolytica* isolates were typed. Download FIG S2, TIF file, 1.1 MB. © Crown copyright 2020. 2020 Crown This content is distributed under the terms of the Creative Commons Attribution 4.0 International license.

### *Lactobacillus* abundance in NS determined by qPCR.

As De Man, Rogosa, and Sharpe (MRS) agar is only semiselective and allows the growth of non-LAB such as *Bacillus* and *Staphylococcus* spp., qPCR was used to estimate the abundance of *Lactobacillus* from the metagenomic DNA extracted from NS. No significant difference in total *Lactobacillus* copy numbers per NS was observed between the two groups with an exception of day 5, when the BT + Mh group had significantly greater abundance of *Lactobacillus* 16S rRNA copies per swab than the Mh group (*P* < 0.05) (Fig. 2B).

### Repetitive sequence-base (GTG)_5_ PCR (rep-PCR) typing of *Lactobacillus* isolates.

An example of (GTG)_5_ fingerprints is shown in Fig. S3. Molecular typing of PCR-confirmed *Lactobacillus* spp. revealed that BT strains were not detected in any NS collected on day −1. In addition, the BT strains were not detected in any Mh calves. On days 3, 5, 7, and 13, respectively, (GTG)_5_ fingerprints of *Lactobacillus* isolates were observed that matched the BT strains, but detection was variable (Table S2). Of the fingerprints that were representative of the BT strains, only Lactobacillus amylovorus 72B was detected in calves beyond day 7, but detection was limited to two calves.

10.1128/mSystems.00629-19.3 FIG S3 A representative example of (GTG)_5_ rep-PCR fingerprints of *Lactobacillus* bacteria isolated from the nasal cavity of challenged dairy calves that received intranasal bacterial therapeutics and *M. haemolytica*. Fragment sizes are indicated on the left of the image, for control markers (1-kb Plus ladder). Strains are listed above each (GTG)_5_ pattern: 103C, 72B, 63A, 86D, 57A, and 3E were bacterial therapeutics described in Materials and Methods. For other strains, animal number with strain ID (N) followed by the replicate number (R) and day of isolation (d) is indicated. Download FIG S3, TIF file, 1.2 MB. © Crown copyright 2020. 2020 Crown This content is distributed under the terms of the Creative Commons Attribution 4.0 International license.

10.1128/mSystems.00629-19.5 TABLE S2 Isolation rate of intranasally inoculated bacterial therapeutic (BT) strains from the nasopharynx of dairy calves (*n* = 12) that received intranasal inoculation of either bacterial therapeutics and *M. haemolytica* (BT + Mh) or *M. haemolytica* only (Mh). Download Table S2, PDF file, 0.2 MB. © Crown copyright 2020. 2020 Crown This content is distributed under the terms of the Creative Commons Attribution 4.0 International license.

### Effects of BT on the composition and diversity of the nasal microbiota.

**FIG 3:**
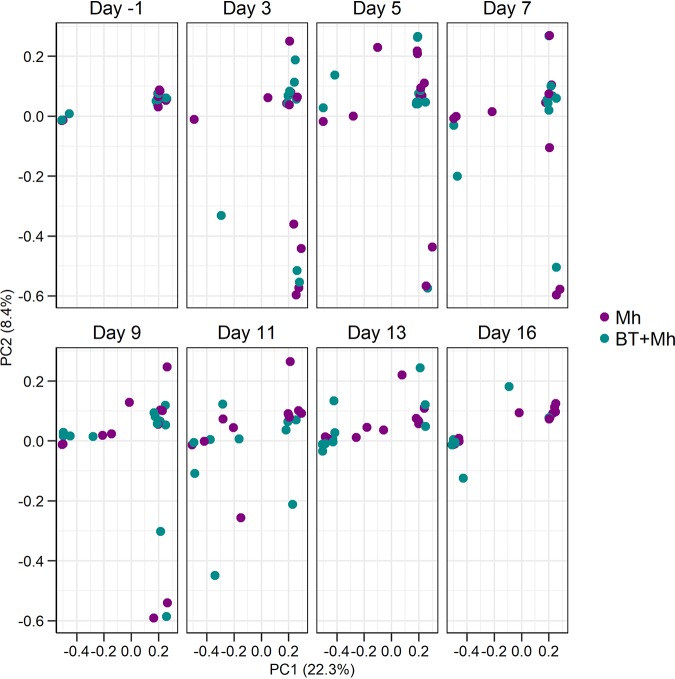
Principal-coordinate analysis (PCA) plots of the Bray-Curtis dissimilarities by treatment and sampling day for the nasal microbiota of calves that received an intranasal inoculation of either bacterial therapeutics and *M. haemolytica* (BT + Mh; *n* = 12) or only *M. haemolytica* (Mh; *n* = 12). The percentages of variation explained by the principal coordinates are indicated on the axes.

In total, 8,869,675 16S rRNA gene sequences (sequences per sample: median = 55,055, minimum = 3,853, and maximum = 91,448) were obtained from 179 NS samples with 4,824 unique archaeal and bacterial operational taxonomic units (OTUs) identified. Permutational multivariate analysis of variance (PERMANOVA) revealed that BT administration (*R*^2^ = 0.011; *P* = 0.02), sampling time (*R*^2^ = 0.052; *P* < 0.01), and replicate (*R*^2^ = 0.028; *P* < 0.01) had significant but relatively minor effects on the microbial structure of the nasal microbiota (Fig. 3). Individual animal had the largest effect (*R*^2^ = 0.247; *P* < 0.001), explaining 24.7% of the variability observed. Although a treatment effect on the microbial community structure was not observed during the first 13 days, there was a larger and significant effect on day 16 (*R*^2^ = 0.15; *P* = 0.01) with 19 OTUs differentially abundant between the two groups (Table S3; false-discovery rate [FDR] < 0.05).

10.1128/mSystems.00629-19.6 TABLE S3 Differentially abundant OTUs at day 16 in the nasopharyngeal microbiota of dairy calves that received intranasal inoculation of only *M. haemolytica* or bacterial therapeutics and *M. haemolytica* (BT + Mh). Download Table S3, PDF file, 0.1 MB. © Crown copyright 2020. 2020 Crown This content is distributed under the terms of the Creative Commons Attribution 4.0 International license.

**FIG 4:**
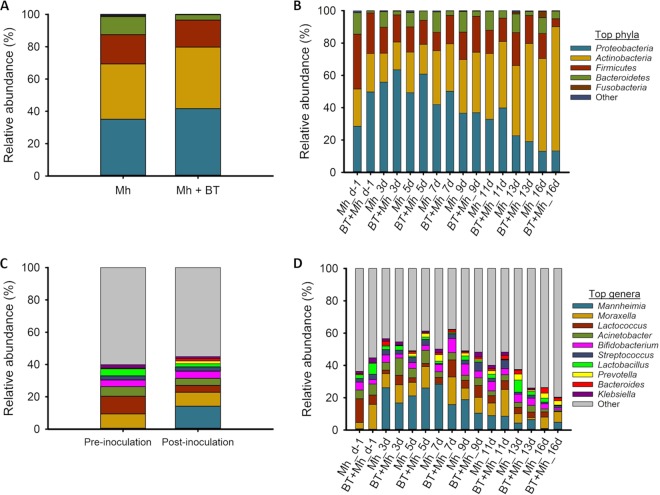
Relative abundance of the 5 most relatively abundant phyla and 10 most relatively abundant genera in the nasal microbiota of calves that received an intranasal inoculation of either bacterial therapeutics and *M. haemolytica* (BT + Mh; *n* = 12) or only *M. haemolytica* (Mh; *n* = 12). (A) Comparison between Mh and BT + Mh calves at the phylum level. (B) Comparison of phyla by groups and sampling day. (C) Comparison before (day −1) and after BT and Mh inoculation (average from days 3 to 16). (D) Comparison of genera by groups and sampling day.

Twenty-five different bacterial phyla were observed among all NS samples, but only *Proteobacteria*, *Actinobacteria*, *Firmicute*s, and *Bacteroidetes* had a relative abundance greater than 0.5% (Fig. 4A). As shown in Fig. 4B, the relative abundance of these phyla changed over time. The relative abundance of *Proteobacteria*, which include the genus *Mannheimia*, increased between day −1 and day 3 and then decreased afterward, whereas the relative abundance of *Actinobacteria* increased throughout the study period to become the most relatively abundant phylum at day 16.

On day −1, of the 10 most relatively abundant genera, *Lactococcus* was the predominant genus with a relative abundance of 10.9%, followed by *Moraxella* (9.0%), Acinetobacter (6.0%), *Lactobacillus* (4.3%), and *Bifidobacterium* (4.0%) among all animals (Fig. 4C). However, after *M. haemolytica* inoculation (i.e., from days 3 to 16), there was a 29-fold increase in the relative abundance of *Mannheimia*, which became the most relatively abundant genus (14.4%; Fig. 4C) across treatments. During that period, the relative abundance of *Lactococcus* declined from 10.9% to 4.23% and *Lactococcus* became the third most abundant genus after *Mannheimia* and *Moraxella*.

A comparison of the 10 most relatively abundant genera between the two groups revealed that the relative abundance of these genera varied by sampling time (Fig. 4D). For example, the nasal microbiota of BT + Mh calves harbored a greater abundance of *Lactococcus* on day 7 but *Lactococcus* was more relatively abundant in the Mh calves on day −1 and day 16 (*P* < 0.05). The relative abundance of *Lactobacillus* did not differ by treatment group at any sampling day except for days 11 and 16, during which Mh calves had a significantly greater relative abundance of *Lactobacillus* than BT + Mh calves (*P* < 0.05). The relative abundance of *Klebsiella* spp. was lower on day 7 but greater on day 9 in BT + Mh calves than in Mh calves (*P* < 0.05). None of the other relatively abundant genera differed significantly between the two groups at any sampling time (*P* > 0.05).

**FIG 5:**
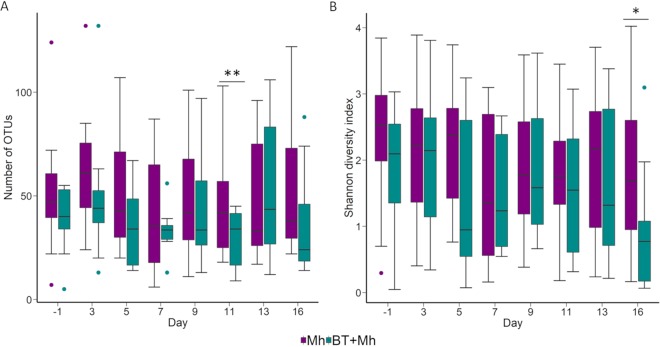
The number of OTUs (A) and Shannon diversity index values (B) of the nasal microbiota of calves that received an intranasal inoculation of either bacterial therapeutics and *M. haemolytica* (BT + Mh; *n* = 12) or only *M. haemolytica* (Mh; *n* = 12). Each box indicates the interquartile range (IQR) (middle 50% of the data), the middle line represents the median value, and the whiskers represent 1.5 times the IQR. Colored dots indicate outliers. Asterisks indicate significant differences between treatments at *P* < 0.05 (*) and *P* < 0.01 (**).

In terms of alpha diversity, the number of OTUs per sample (richness) was affected by both treatment (*P* = 0.04) and time (*P* < 0.01) (Fig. 5A). The Mh calves had a significantly higher number of OTUs on day 11 than the BT + Mh calves (*P* < 0.01). No difference was observed between groups for the Shannon diversity index except on day 16, with greater diversity in the Mh calves (*P* = 0.04; Fig. 5B).

### Effects of BT on the recursive structure of causal relationships among the 10 most relatively abundant genera in the nasal microbiota.

**FIG 6:**
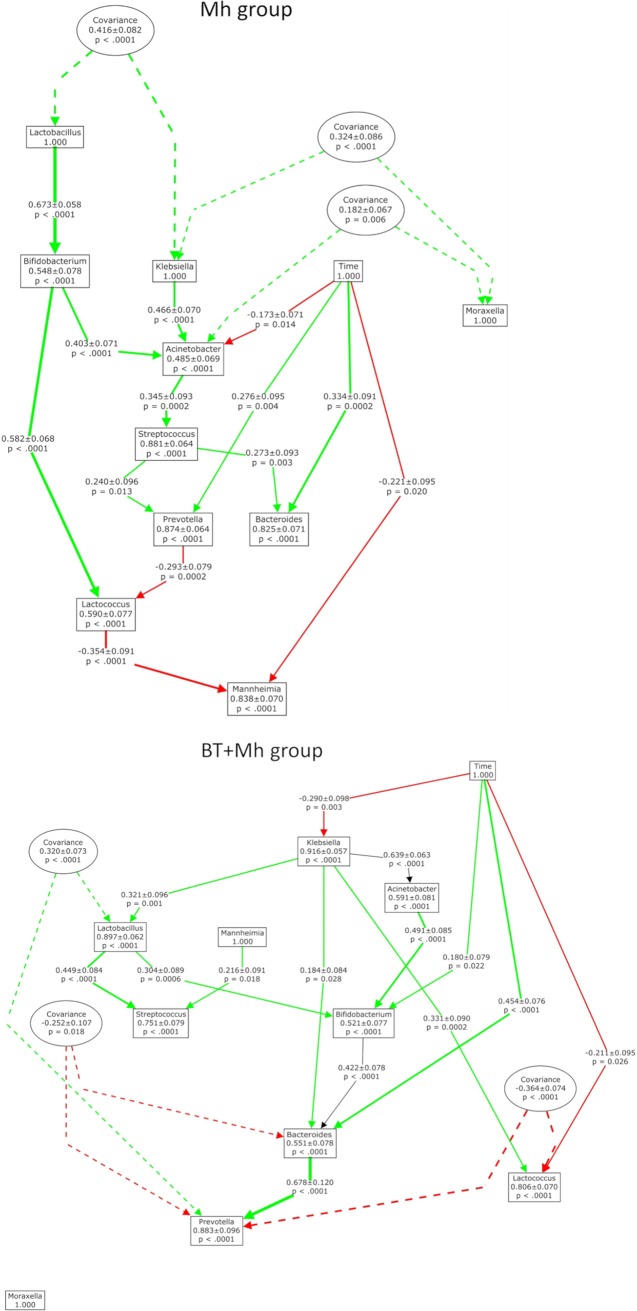
Path diagram of models showing the recursive structure of causal relationships among the 10 most abundant genera in the nasal microbiota of calves that received an intranasal inoculation of either bacterial therapeutics and *M. haemolytica* (BT + Mh; *n* = 12) or only *M. haemolytica* (Mh; *n* = 12). Variances of measured variables (relative abundances of genera and time) with standard errors and *P* values are shown in squares. Causal relationships that are implied by the model are shown as solid lines with arrows that indicate the direction of causation. Causal paths are labeled with the standardized path coefficients, standard errors, and *P* values. Covariance terms are shown in ovals with dashed arrows between the variables that covary. The green line represents the positive effect, whereas the red line represents the negative effect. The thickness of the solid line represents the strength of the effect.

To evaluate whether the causal relationships among genera in nasal microbiota changed in response to the intranasal inoculation of BT, path analysis was performed on the relative abundance data of the 10 most abundant genera from all sampling days. The matrices of Spearman correlation coefficients that were used as the basis for path analysis are provided as Tables S4 and S5. Two path models (Fig. 6; Table S6) were selected based on the model fit statistics (Table S6). Based on the values of the squared multiple correlation, *R*^2^, model 1, constructed based on the data from the Mh group, explained 11.90 to 51.47% of the variance in relative abundances of the endogenous genera, while model 2 (BT + Mh group) explained 8.43 to 44.94% of the variance in relative abundances of the endogenous genera (Table S6).

10.1128/mSystems.00629-19.7 TABLE S4 Spearman rank-based correlation coefficients of relative abundance of top 10 most abundant genera within the nasal microbiota of calves (*n* = 12) that received an intranasal inoculation of *M. haemolytica*. Download Table S4, PDF file, 0.1 MB. © Crown copyright 2020. 2020 Crown This content is distributed under the terms of the Creative Commons Attribution 4.0 International license.

10.1128/mSystems.00629-19.8 TABLE S5 Spearman rank-based correlation coefficients of relative abundance of top 10 most abundant genera within the nasal microbiota of calves (*n* = 12) that received an intranasal inoculation of bacterial therapeutics and *M. haemolytica*. Download Table S5, PDF file, 0.1 MB. © Crown copyright 2020. 2020 Crown This content is distributed under the terms of the Creative Commons Attribution 4.0 International license.

10.1128/mSystems.00629-19.9 TABLE S6 Path models fitted to data corresponding to statistics that are Schwarz’s (Bayesian) information criteria of three path models fitting subsets of experimental data. Download Table S6, PDF file, 0.4 MB. © Crown copyright 2020. 2020 Crown This content is distributed under the terms of the Creative Commons Attribution 4.0 International license.

Path modeling (Fig. 6; Table S6) indicated a distinct causal relationship among the 10 most relatively abundant genera of the nasal microbial community between Mh and BT + Mh groups. The relative abundances of seven genera including Acinetobacter, *Bacteroides*, *Bifidobacterium*, *Lactococcus*, *Mannheimia*, *Prevotella*, and *Streptococcus* were endogenous (variance explained by factors inside the model such as relative abundance of other genera and time) while the relative abundances of three genera (*Klebsiella*, *Lactobacillus*, and *Moraxella*) were exogenous (variance explained by unmeasured factors outside the model) in the Mh group. Within the nasal microbial community of Mh calves, 16.2% of the variance in the relative abundance of *Mannheimia* was explained by a combination of other genera and sampling time (Table S7). For example, the genus *Lactobacillus* was positively linked with the relative abundance of *Bifidobacterium*, which in turn promoted *Lactococcus. Lactococcus*, on the other hand, was negatively associated with the relative abundance of *Mannheimia*. In addition, *Prevotella* was predicted to have an indirect positive effect on *Mannheimia* by negatively impacting the *Mannheimia* inhibitor *Lactococcus.* The relative abundance of *Prevotella* was predicted to be positively affected by *Streptococcus*, Acinetobacter, and *Klebsiella* and time.

10.1128/mSystems.00629-19.10 TABLE S7 Squared multiple correlations in path models fitted to data from the relative abundance of top 10 genera of nasal microbiota in calves that received an intranasal inoculation of either only *M. haemolytica* (model 1) or bacterial therapeutics and *M. haemolytica* (model 2). Download Table S7, PDF file, 0.2 MB. © Crown copyright 2020. 2020 Crown This content is distributed under the terms of the Creative Commons Attribution 4.0 International license.

Interestingly, the path model for BT + Mh calves differed from that for Mh calves, indicating that causal relationships between the 10 most abundant genera were likely altered by BT administration. *Mannheimia* was found to be an exogenous variable in the BT + Mh group whose relative abundance was not affected by any of the other genera or time. The relative abundance of *Moraxella* was also an exogenous variable in the BT + Mh group. Unlike the Mh group, no associations between *Lactococcus*, *Prevotella*, and *Mannheimia* were predicted. Of note, both *Lactobacillus* and *Mannheimia* were predicted to be positively associated with *Streptococcus.* The relative abundance of *Klebsiella* was predicted to decline over time in the BT + Mh group but not in the nasal microbiota of Mh calves.

### Effects of BT on the composition and diversity of the tracheal microbiota.

**FIG 7:**
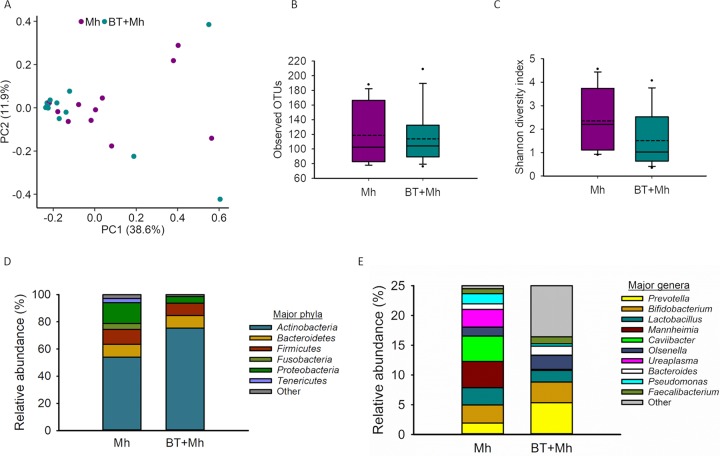
Description of the tracheal microbiota of calves that received an intranasal inoculation of either bacterial therapeutics and *M. haemolytica* (BT + Mh) or only *M. haemolytica* (Mh). (A) Principal-coordinate analysis plots of the Bray-Curtis dissimilarities; percentages of variation explained by the principal coordinates are indicated on the axes. (B) Box plots of observed OTUs. (C) Shannon diversity index. (D) The six most relatively abundant phyla. (E) The 10 most relatively abundant genera.

For the transtracheal aspiration (TTA) samples collected on day 16, there were 1,585 OTUs identified among 1,895,098 sequences (sequences per sample: median = 78,297, minimum = 25,968, maximum = 141,708). PERMANOVA indicated that the tracheal microbiota structure on day 16 was not significantly affected by BT treatment (*R*^2^ = 0.065; *P* = 0.10) or replicate (*R*^2^ = 0.071; *P* = 0.07) (Fig. 7A).

The richness (number of OTUs) and diversity (Shannon diversity index) of the tracheal microbiota also did not differ between the BT + Mh and Mh calves (*P* > 0.05; Fig. 7B and C).

Twenty-nine bacterial phyla were detected among the TTA samples, with six having a relative abundance greater than 0.5% across treatments: *Actinobacteria* (65.6%), *Proteobacteria* (9.8%), *Firmicutes* (9.7%), *Bacteroidetes* (9.2%), *Fusobacteria* (2.1%), and *Tenericutes* (1.5%) (Fig. 7D). The Mh calves had a significantly greater relative abundance of *Proteobacteria* and *Fusobacteria* than BT + Mh calves (*P < *0.05). *Actinobacteria*, however, tended (*P* = 0.07) to be more abundant in the BT + Mh group.

A total of 392 genera were identified within the tracheal microbiota; however, only 13 had an overall relative abundance greater than 0.5%. Of the 10 most relatively abundant genera, only three (*Mannheimia*, *Lactobacillus*, and *Prevotella*) were also among the most relatively abundant genera detected in the nasal microbiota (Fig. 7E). Of note, the relative abundance of *Mannheimia* (*P* < 0.0001) and *Pseudomonas* (*P* < 0.01) was significantly greater in Mh calves than in BT + Mh calves but the relative abundance of *Lactobacillus* did not differ between these two groups. Though relative abundance was found to be highly variable, the mean abundances of *Ureaplasma* and *Caviibacter* genera were also significantly greater in the Mh group than in the BT + Mh group (*P* < 0.001).

### Effects of bacterial therapeutics on serum cytokine concentrations.

**FIG 8:**
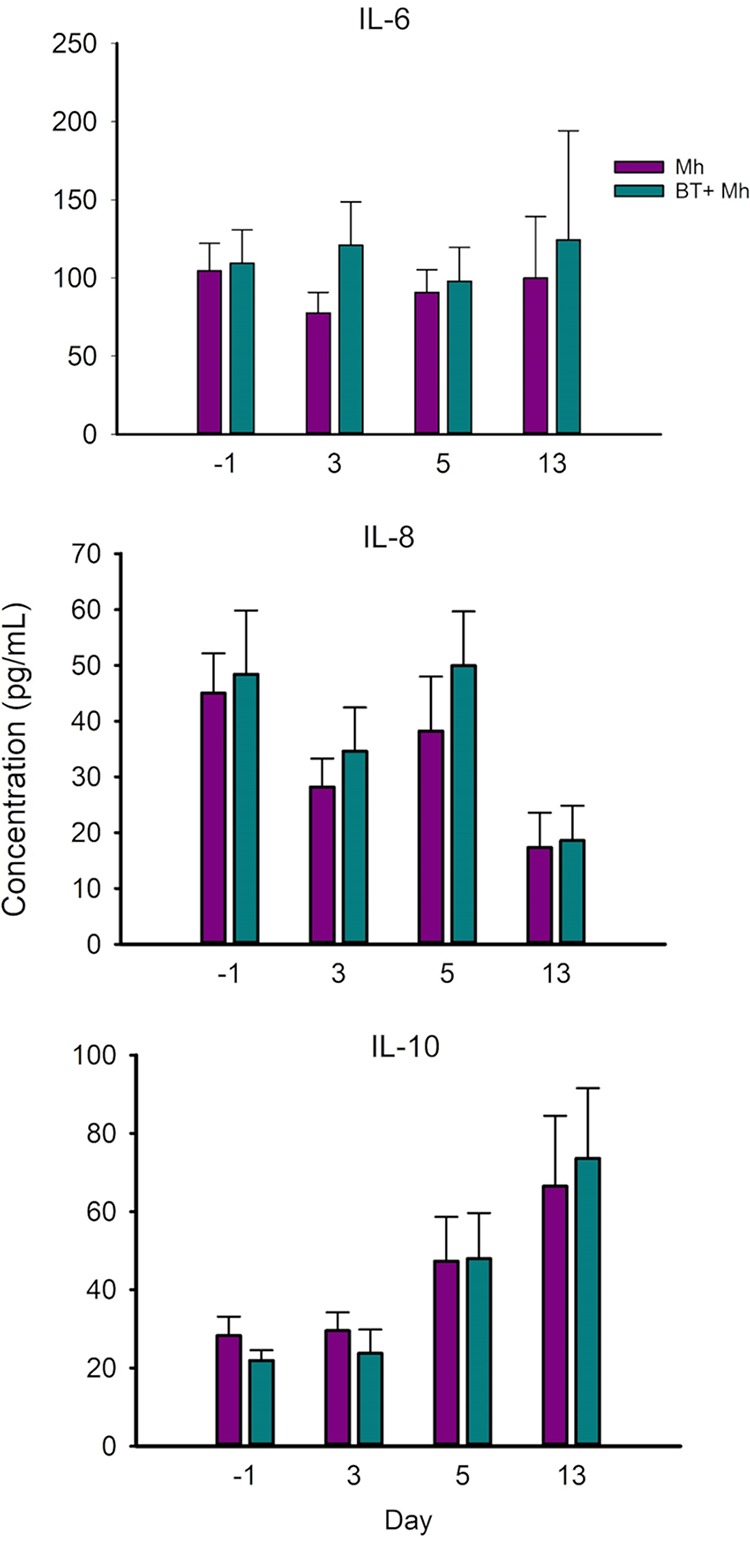
Serum cytokine (IL-6, IL-8, and IL-10) concentrations by sampling day in dairy calves that received an intranasal inoculation of either bacterial therapeutics and *M. haemolytica* (BT + Mh; *n* = 12), or only *M. haemolytica* (Mh; *n* = 12).

No difference in concentrations of serum cytokines IL-6, IL-8, and IL-10 was observed between the two groups at any sampling times (*P* > 0.05) (Fig. 8). Tumor necrosis factor alpha (TNF-α) was not detected from the serum samples of any calf at any sampling times (detection limit of the enzyme-linked immunosorbent assay [ELISA] kit used was 0.1 ng/ml).

## DISCUSSION

Increasing evidence indicates that commensal members of the bovine respiratory microbiota have competitive relationships with opportunistic pathogens and may be involved in a microbiota-mediated defense against respiratory infection (18, 20, 22). Therefore, we recently screened commensal bacteria (*n* = 178) isolated from the respiratory tract of healthy cattle for the development of intranasal BT to mitigate the BRD pathogen *M. haemolytica*, as an alternative to metaphylactic antimicrobial use. By ranking BT candidates based on *in vitro* inhibition of *M. haemolytica*, as well as epithelial cell colonization and immunomodulation, we identified six *Lactobacillus* strains for evaluation in cattle. In the study reported here, we further evaluated the *in vivo* potential of these selected strains when administered intranasally in young dairy calves that were subsequently challenged with *M. haemolytica*.

Intranasal administration of the BT cocktail did not adversely affect calf health, implying that the dose of inoculation tested was tolerated by young dairy calves. The *Lactobacillus* strains we selected for nasal inoculation upregulated (moderate-level) gene expression of IL-6 and IL-8 in bovine turbinate cells *in vitro* (22). However, in the present study, there were no significant differences in serum concentrations of IL-6, IL-8, and IL-10 between Mh and BT + Mh calves. Unlike the previous *in vitro* experiment where these selected *Lactobacillus* strains were cocultured with turbinate cells without the presence of *M. haemolytica*, in the present study, all calves were challenged with *M. haemolytica* after BT inoculation. There were no groups of calves that received only BT nor a control group that received neither BT nor *M. haemolytica*. Therefore, it was difficult to extrapolate the findings from the *in vitro* experiment into this challenge study.

The BT cocktail reduced nasal colonization by *M. haemolytica*, which was expected based on our previous *in vitro* studies (19, 22). Indeed, all six *Lactobacillus* strains inoculated have been shown to inhibit *M. haemolytica* adherence to bovine turbinate cells by 32% to 78% (22). Furthermore, these strains produced 80 to 142 mM lactic acid after 24 h of incubation, which is sufficient to inhibit the growth of *M. haemolytica* (22). Finally, genome sequencing showed that two of the strains (*L. amylovorus* 72B and Lactobacillus paracasei 3E) (22, 23) encoded bacteriocins which have bactericidal or bacteriostatic activity (24, 25). Therefore, the reduction in *M. haemolytica* colonization could have partially resulted from competition for adherence to the nasal mucosa or direct inhibition of *M. haemolytica*.

It is also possible that the BTs modified the nasal microbiota in a way that conferred increased colonization resistance to *M. haemolytica*. A previous study showed that microbiota manipulation using probiotic *Lactobacillus* strains reduced the colonization of vancomycin-resistant *Enterococcus* (VRE) in challenged mice (26). The authors proposed that the compositional alteration observed from phylum to species level by probiotic strains may have contributed to decreased VRE colonization. The altered microbial community structure observed in our study, as well as differences observed in the causal relationships between the 10 most relatively abundant genera of nasal microbiota between BT + Mh and Mh treatments, suggests that microbiota-mediated colonization resistance may also have resulted in increased resistance to *M. haemolytica* colonization and proliferation. This may explain why there was a reduction in colonization of *M. haemolytica* from days 3 to 7, while *Lactobacillus* was only greater in BT + Mh calves on day 5. Finally, reduced nasal colonization by *M. haemolytica* could also be a consequence of the activation of the mucosal innate immune system by BT. Indeed, *Lactobacillus* strains can beneficially modulate the immune response of the host and confer resistance against respiratory pathogens (27). For example, nasal administration of Lactobacillus rhamnosus in mice was able to modulate the immune response triggered by Toll-like receptor 3 (TLR3)/RIG-I activation in the respiratory tract and increased the resistance of mice to a challenge with respiratory syncytial virus (27). Unfortunately, as we did include a positive-control group of calves receiving only the BT cocktail, it was not possible to investigate the effect of BT on the mucosal immunity.

A single administration of BTs was performed to align with management strategies of calves, which are typically handled and processed only once early in life. As observed by qPCR, a transient increase in the number of *Lactobacillus*-specific 16S rRNA gene copies occurred but only on day 5. This implies that the majority of strains in the BT mixture did not colonize the nasal cavity of calves for more than 2 days after inoculation. However, Lactobacillus buchneri 86D and *L. amylovorus* 72B were isolated from two calves on days 7 and 13, respectively, indicating successful colonization by these BT strains in these calves. The differences observed between strains and the variation in calf colonization are difficult to explain but may be due to several factors. First, the method of delivery may have resulted in colonization variation as phosphate-buffered saline (PBS) suspensions rapidly dissipated upward and downward of the application site. Improved delivery methods may thus reduce variability. Second, the source of calves may also have resulted in different microbiotas between calves and, as a result, altered resistance to BT colonization. In support of this, most of the variability observed in the composition of the nasal microbiota was explained by differences among calves. This is not surprising, as the calves originated from different farms, and it has previously been shown that the dam vaginal microbiota influences the respiratory microbiota of calves (28). In addition, the presence of species similar to those inoculated may limit colonization of probiotics. For example, indigenous Bifidobacterium longum in the human gut was shown to prevent colonization of the exogenous B. longum AH1206 strain (29). Zmora et al. (30) also observed that the successful colonization of probiotic strains in the human gut was negatively affected by the presence and abundance of indigenous members of the same genera to which the probiotic strains belonged. Indeed, we detected *Lactobacillus* spp. in calves prior to inoculation via qPCR. Thus, enhanced colonization may be achieved if the BTs are delivered soon after birth and before the establishment of the respiratory microbiota. Finally, the BT strains originated from postweaned feedlot beef steers (22). As the nasal microbiota can differ between preweaned dairy calves (31) and postweaned feedlot beef cattle (32), it is possible that these BT strains were better adapted to beef cattle.

Despite colonization being transient, an impact of BT on the nasal microbial community was observed even after the majority of BT strains were no longer detectable in the NS. Although probiotics are frequently reported as poorly established members of the gut microbiota (33, 34), the effect of probiotics on the gut microbiota structure and diversity has been reported to be evident from 2 weeks up to 5 months after cessation of probiotic consumption (30, 35, 36). The underlying mechanisms by which the BT strains may modulate the nasal microbiota long-term are challenging to explain. However, one of the mechanisms could be associated with the impact of introducing BT strains on the microbial interaction network among members of the nasal microbiota. Microbial communities in most niches form complex ecological interaction webs, and such interactions are important in maintaining microbiota homeostasis and symbiotic relationships between microbes and host (37). Here, we observed significantly different structures of causal relationships between the 10 most relatively abundant genera as evident by the path models. The biological significance of these causal relationship structures observed remains to be determined. However, it has been recently suggested that probiotic bacteria may promote intestinal microbiota homeostasis by enhancing species-species interactions and increasing the number of connectors and/or module hubs within the network (38). Hence, it is tempting to speculate that BT inoculation may have also positively affected the microbial relationship and thereby promoted microbial homeostasis, which may have contributed to reduced microbial diversity and richness observed in the last week of study.

Path analysis models can help to predict potential mutualistic and antagonistic interactions among bacterial species (39). In the present study, the relative abundance of *Mannheimia* in Mh calves was predicted by path modeling to be negatively affected by *Lactococcus* and time (direct) and *Bifidobacteria* and *Lactobacillus* (indirect). The fact that *Lactococcus* could antagonize *M. haemolytica* is in agreement with a previous study (21) which showed that *Lactococcus* was more abundant in the lower respiratory tract of healthy feedlot cattle than cattle with BRD (which had a higher abundance of *M. haemolytica*). Furthermore, we have previously shown that Lactococcus lactis could inhibit the growth of *M. haemolytica in vitro* (19). *Lactococcus* spp. were relatively abundant among all calves (10.9%) on day −1. This indicates that species within *Lactococcus* may have a role in maintaining respiratory homeostasis in the early life of calves. Surprisingly, in the BT + Mh group, *Mannheimia* was found to be an exogenous variable whose relative abundance was not affected by the other tested genera, including *Lactobacillus*. This finding is difficult to explain, and further research should be conducted to better understand how the *Lactobacillus* strains that we inoculated affected communication and microbial networks among nasal bacteria. It is possible that limiting the path models to the 10 most relatively abundant genera excluded minor genera that could have affected *Mannheimia* endogenously in the BT + Mh calves. Regardless, our study showed that a single dose of BTs could affect the respiratory microbiota of calves, highlighting that BTs may be an effective method to modulate the respiratory microbiota. Thus, further research on the method and timing of administration, as well as additional BT species, is warranted to enhance pathogen resilience of the bovine respiratory microbiota.

To our best knowledge, this is the first study to profile the tracheal microbiota in young dairy calves (<5 weeks of age). Despite being removed from the dam and dairy farm within 3 h postbirth and housed in a clean and controlled environment, these calves harbored a diverse (393 different genera identified) and rich tracheal microbial community. 16S RNA gene sequencing of TTA samples collected on day 16 revealed that the tracheal microbiota contained more phyla than the nasal cavities (29 versus 26), and only 3 out of the 10 most abundant genera were shared between these two anatomical sites. This suggests that a self-sustaining and established microbiota is present in the lower respiratory tract of newborn calves. Comparing the microbial profiles between the BT + Mh and Mh groups, no significant differences were observed for both the beta and alpha diversity indices. However, there were alterations in the relative abundance of specific phyla and certain genera in the BT + Mh group relative to the Mh group.

The *M. haemolytica* challenge design used in the present study was a colonization model and not a disease model, explaining the absence of abnormal clinical signs during the study period. To experimentally induce disease (i.e., pneumonia), *M. haemolytica* has to be inoculated directly into the lower respiratory tract (bypassing the upper respiratory tract) (40) or should be inoculated after a viral infection (i.e., bovine herpesvirus 1) (41, 42). Ten calves nevertheless had lung lesions, and *M. haemolytica* was found to be more abundant in the Mh than in the BT + Mh group. Therefore, it is possible that some *M. haemolytica* bacteria reached the lower respiratory tract and created lesions, especially in the Mh calves. It is interesting to speculate that the BTs reduced colonization of *M. haemolytica* in the upper respiratory tract of BT + Mh calves, thereby reducing the amount of *M. haemolytica* translocating to the lungs. It is generally accepted that proliferation of BRD pathogens is a prerequisite to lung infection (43). Indeed, some parenteral metaphylactic antibiotics given to feedlot cattle upon arrival at the feedlot have been shown to reduce the prevalence of *M. haemolytica* in the nasopharynx of calves, which may be related to their efficacy (44). Thus, should BTs reduce proliferation or the abundance of *M. haemolytica* in the upper respiratory tract, they may also limit translocation of the pathogen to the lungs. A next logical step would be to investigate if the nasal inoculation with BTs can prevent the development of BRD due to *M. haemolytica*.

In summary, a single-dose inoculation of intranasal BTs developed from six *Lactobacillus* strains originating from the bovine respiratory microbiota was able to reduce nasal colonization by *M. haemolytica* in experimentally challenged dairy calves. Administration of BTs also altered the recursive structure of causal relationships among the 10 most relatively abundant genera within the nasal microbiota of calves. A lower relative abundance of *M. haemolytica* in the trachea of calves that received BT was observed at the end of the study period. Finally, the administration of BT did not stimulate a systemic immune response based on the cytokines tested. Overall, the results of this study, for the first time, demonstrated the potential use of intranasal BTs to mitigate the BRD pathogen *M. haemolytica* in cattle. Further research should be conducted to investigate if intranasal BT inoculation can prevent respiratory disease, which is highly relevant to the cattle industry.

## MATERIALS AND METHODS

### Ethics statement.

This study was conducted in strict accordance with the recommendations of the Canadian Council of Animal Care (45). The research protocol was reviewed and approved by the University of Calgary Veterinary Sciences Animal Care Committee (AC17-0003).

### Animals and husbandry.

Twenty-four Holstein bull calves from six different local dairy farms were used in the study. Calves were separated from the dam immediately after birth to prevent suckling and were fed 3 liters of a colostrum replacer (Calf’s Choice Total HiCal; SCCL, Saskatoon, SK, Canada) within 3 h of birth to provide adequate passive immune transfer and reduce variability among calves. Calves were then transported within 3 h to a research facility, housed individually, and fed another 3 liters of colostrum replacer. Navels were sprayed with 7% iodine solution daily for 3 day following birth. A milk replacer enriched with a colostrum substitute (20% of total solids; Hical; SCCL) was fed to the calves twice daily until 7 days of age. From 7 days onward, calves were fed only milk replacer and the amount of milk replacer was adjusted based on their body weight. Clean water was available at all times, and starter grain was fed *ad libitum* after 14 days of age.

### Study design.

The challenge study was repeated twice at the same research facility with 12 calves per replicate. The second replicate started approximately 1 month after the first replicate was completed. The schematic of experimental design and of sampling regimen is presented in Fig. 1. After an acclimatization period (at least 7 days), all calves were sampled using nasal swabs (NS) to evaluate their *M. haemolytica* and LAB status (i.e., positive or negative; day −1). On day 0, calves were blocked by age and farm of origin, as well as *M. haemolytica* and LAB culture results obtained on day −1. They were then randomly assigned into either BT + Mh or Mh groups (*n* = 6 per group). On day 1, calves in the BT + Mh group were administered in each nasal cavity 3 ml of phosphate-buffered saline (PBS) containing a multistrain cocktail of 6 *Lactobacillus* strains in equal concentrations (1 × 10^9^ CFU ml^−1^), whereas the Mh group was administered 3 ml of PBS without bacteria. On day 2, all calves were challenged with 1 × 10^8^ CFU ml^−1^ of *M. haemolytica* by administering 3 ml into each nasal cavity. This dose has previously been established to represent a colonization model for *M. haemolytica* without inducing bronchopneumonia (46, 47). The intranasal delivery of PBS with or without bacteria was applied to the ventral meatus, 4 to 5 cm from the nostril entrance, using a sterile 12-ml syringe fitted with a 9-cm-long sterile tube (Fig. 1).

During the study period, calves were monitored daily by an experienced bovine veterinarian (E.T.) for the following clinical signs: demeanor, appetite, rectal temperature, respiratory rate, nasal and ocular discharge, and presence of abnormal sounds at lung auscultation. Nasal swab samples were collected on days −1, 3, 5, 7, 9, 11, 13, and 16, and blood samples were collected on days −1, 3, 5, and 13 (as shown in Fig. 1). Calves were euthanized on day 16 and sampled immediately by transtracheal aspiration (TTA), and their lungs were evaluated for gross lesions at necropsy.

### Sample size calculation.

The sample size (*n* = 12 calves per treatment group) was calculated to detect, at least, a 50% difference in the proportion of calves positive for *M. haemolytica* 9 days after challenge between the BT + Mh and Mh groups. For this calculation, α and β were set at 0.05 and 0.20, respectively (two-sided test).

### Preparation of the nasal inoculum.

The *M. haemolytica* (Guelph 1) strain used for the challenge was isolated from a BRD-affected beef calf in a feedlot and identified as serotype 1 (48). This strain was plated onto brain heart infusion (BHI; Oxoid, Nepean, ON, Canada) agar, and a single colony was inoculated into 50 ml of BHI broth, followed by incubation with shaking at 37°C for 18 h. The culture was then centrifuged at 3,000 × *g* for 20 min, and the cell pellet was resuspended in 10 ml of PBS and washed twice using PBS. The cell pellet was diluted in PBS to obtain a final suspension containing 1 × 10^8^ CFU ml^−1^ for administration to calves.

The BT cocktail included the following 6 *Lactobacillus* strains: *L. amylovorus* (72B), *L. buchneri* (63A and 86D), Lactobacillus curvatus (103C), and *L. paracasei* (3E and 57A), each at a concentration of 1 × 10^9^ CFU ml^−1^ (Fig. 1). These *Lactobacillus* isolates were plated onto *Lactobacillus* De Man, Rogosa, and Sharpe (MRS) agar (Dalynn Biologicals, Calgary, AB, Canada) and incubated for 48 h at 37°C in 10% CO_2_. One day prior to nasal inoculation, a single colony of each strain was inoculated individually into 5 ml Difco lactobacillus MRS broth (BD, Mississauga, ON, Canada) and incubated at 37°C with agitation at 200 rpm. After 18 h of incubation, each bacterial culture was centrifuged at 7,600 × *g* for 10 min, the supernatant was discarded, and the pellet was resuspended with PBS. Aliquots of each strain were then mixed together to achieve a target concentration of 1 × 10^9^ CFU ml^−1^ to serve as the BT cocktail for administration. Inoculants for calves were prepared on the day of administration. The concentrations of *M. haemolytica* and individual BT strains (immediately prior to mixing as a cocktail) were confirmed by plating and enumeration.

### Sampling and processing of NS.

Nasal swab samples were collected using 10-cm-long unprotected swabs, which had tips flocked with soft nylon fiber (flocked swab; 480C ESwa Liquid Amies Collection and Transport System; Copan, Murrieta, CA, USA). Two NS were collected from each calf (one from each nostril). Before sampling, each nostril was wiped with a clean paper towel to remove any debris or nasal discharge. Immediately after sample collection, tips of the swabs were broken and placed in 1 ml of sterile Amies transport medium. The swabs were stored at 4°C overnight and processed the following morning.

For processing, the tip was cut from the swab and placed into 0.7 ml of BHI broth. The transport tube containing the Amies medium was then centrifuged (2,000 × *g*, 5 min), and 75% of the supernatant was discarded. The pellet was resuspended with the remaining supernatant, transferred to the BHI tube containing the rayon tip of the swab, and vortexed for 30 s.

### Isolation and enumeration of *M. haemolytica* from NS.

For enumeration of *M. haemolytica*, a 100-μl aliquot of the swab-BHI suspension was serially diluted in PBS. The dilutions were plated onto tryptic soy agar (TSA) containing 5% sheep blood supplemented with 15 μg ml^−1^ of bacitracin (to limit the growth of Gram-positive bacteria [49] [Dalynn Biologicals]) and incubated overnight at 37°C. Each swab from the right and left nasal cavities was processed separately, and bacterial counts for each animal were obtained by averaging CFU from the two swabs. Up to three colonies displaying typical morphology indicative of *M. haemolytica* (white-gray, round, medium-sized, nonmucoid, exhibiting β-hemolysis) were substreaked onto TSA with 5% sheep’s blood and incubated overnight at 37°C. Three glycerol stocks (1.2 ml; BHI: glycerol, 80%:20%) for each *M. haemolytica* isolate from these plates were prepared and immediately stored at −80°C for further analyses (i.e., pulsed-field gel electrophoresis; see below). In addition, a loop of each isolate was also stored in 100 μl Tris-EDTA (TE) at −80°C for PCR confirmation.

### PCR identification and PFGE typing of *M. haemolytica*.

DNA was extracted from each *M. haemolytica* isolate using heat lysis at 98°C for 3 min and used for PCR confirmation of *M. haemolytica* as described by Klima et al. (14). Afterward, a subset of PCR-confirmed *M. haemolytica* isolates (*n* = 44) was randomly selected (per isolate per animal per sampling time point) and typed by pulsed-field gel electrophoresis (PFGE) to determine relatedness with *M. haemolytica* Guelph 1. PFGE typing was performed as described previously (13), and PFGE profiles were analyzed using BioNumerics 7.6 (Applied Maths, Inc., Austin, TX, USA).

### Isolation of LAB from NS.

MRS agar, which is semiselective for LAB, was used to isolate *Lactobacillus* spp. A 100-μl aliquot from each NS was serially diluted, plated on MRS agar, and incubated for 48 h in a 10% CO_2_-enriched environment at 37°C. Up to 10 colonies from each animal were subcultured on MRS agar. The subcultures were then processed for glycerol stocks (MRS: glycerol, 80%:20%) and TE stocks as described for *M. haemolytica*. DNA isolation from TE stocks was used for PCR confirmation and subtyping (see below). Each swab from the right and left nasal cavities was processed separately, and bacterial counts for each animal were obtained by averaging CFU from the two swabs.

### PCR identification and rep-PCR typing of *Lactobacillus* isolates.

Because some non-LAB species such as *Bacillus* and *Staphylococcus* spp. grow on MRS agar (32), isolates within *Lactobacillus* were first identified using a *Lactobacillus* genus-specific PCR assay. Briefly, an aliquot of 20 μl of TE stock was heat lysed at 98°C for 3 min, and the lysate was used to identify *Lactobacillus* isolates using genus-specific primers according to the work of Dubernet et al. (50). The remaining TE stock from isolates that tested positive as *Lactobacillus* was processed for genomic DNA extraction using a Qiagen DNeasy tissue kit (Qiagen Inc., Mississauga, ON, Canada) as described by Holman et al. (16).

The extracted genomic DNA was used for molecular typing using the repetitive sequence-base (GTG)_5_ PCR (rep-PCR) method. The rep-PCR was performed using the (GTG)_5_ primer (51). Briefly, the PCR mixture (20 μl) contained 10 μl of Hotstart master mix (Qiagen Inc.), 2 μM primer, 2 μl of genomic template DNA, and 6 μl of H_2_O. DNA fragments were amplified using an Eppendorf Mastercycler Pro thermal cycler (Eppendorf Canada, Mississauga, ON, Canada) using the following PCR conditions: initial denaturation at 94°C for 5 min, followed by 35 cycles consisting of denaturation at 94°C for 30 s, annealing at 40°C for 60 s, and extension at 72°C for 10 min, and a 10-min final extension step at 72°C.

The (GTG)_5_-PCR amplicons were analyzed on 1.5% (wt/vol) SeaKem gold agarose (Lanza, Rockland, ME, USA) gels in Tris-borate-EDTA buffer (ThermoFisher Scientific, Ottawa, ON, Canada). A 1-kb Plus DNA ladder (Invitrogen, Carlsbad, CA, USA) was used as a molecular size marker according to the manufacturer’s directions. The electrophoresis was performed using a Bio-Rad PowerPac 300 (Bio-Rad Laboratories, Redmond, WA) at 4.0 V/cm for 16 h at 4°C. Gels were stained with ethidium bromide (5 μg/ml; Bio-Rad) and visualized under UV light using the ChemiDoc MP imaging system (Bio-Rad).

### Blood sample collection and processing.

Blood (4 ml) was collected from the jugular vein using plain collection tubes (BD Vacutainer blood collection tubes; Becton, Dickinson and Company, Franklin Lakes, NJ). Blood samples were processed within 6 h and centrifuged at 2,000 × *g* for 10 min at room temperature. Resulting sera were then stored at −80°C until further analysis.

### Quantification of cytokines in serum using ELISA.

The concentrations of the cytokines IL-6, IL-8, IL-10, and TNF-α from serum samples collected on days −1, 3, 5, and 13 were determined using commercially available ELISA kits (bovine IL-4, IL-6, or TNF-α screening set [Thermo Scientific]; human CXCL8/IL-8 DuoSet [R & D Systems, Wiesbaden, Germany]; bovine IL-10 ELISA kit [MyBioSource Inc., San Diego, CA, USA]) according to the manufacturer’s instructions.

### Transtracheal aspiration sampling and processing.

Transtracheal aspirations were carried out as described by Timsit et al. (12) on each calf immediately after euthanasia to characterize the tracheal microbiota. Briefly, 50 ml of sterile saline (0.9% NaCl) was introduced in a 75-cm-long transtracheal catheter (Centracath; Vygon, Ecouen, France) using a 50-ml syringe. Immediately after injection, gentle suction was provided by withdrawing the plunger. On average, 5 to 10 ml of tracheal fluid was recovered and immediately placed into empty sterile tubes. Samples were placed on ice and stored at −80°C within 12 h of collection.

### Postmortem examination.

As part of the safety assessment for BT inoculation, all calves were euthanized at the end of the experiment (day 16) and necropsied by a board-certified veterinary pathologist at the Diagnostic Services Unit of the University of Calgary. Each lung was removed and scored for macroscopic lung lesion (i.e., lung consolidation).

### DNA extraction from NS and TTA samples and 16S rRNA sequencing.

Total DNA was extracted from the remaining NS suspensions using a method with enzyme and mechanical cell disruption, as previously detailed (16). For DNA extraction from TTA samples, 2 ml of each TTA sample was transferred into a 2-ml centrifuge tube and centrifuged at 15,000 × *g* for 3 min. The supernatant was discarded, and DNA was extracted from the pellet using the same method as for NS.

The nasal and tracheal microbiota were characterized through sequencing of the V4 hypervariable region of the 16S rRNA gene using the MiSeq reagent kit v2 (500 cycles) and an Illumina MiSeq (Illumina, San Diego, CA, USA) as previously described (52). The 16S rRNA gene sequences were processed using DADA2 v. 1.8.0 (53) in R v. 3.5.0. Briefly, primer sequences were removed, forward and reverse reads were truncated at 220 bp, and reads with a maximum number of expected errors greater than 2 were eliminated. The forward and reverse reads were then merged, and chimeric sequences were removed. The RDP naive Bayesian classifier (54) and the SILVA SSU database release 132 (55) were used to assign taxonomy to each merged sequence, referred to here as operational taxonomic units (OTUs), at 100% similarity. The Shannon diversity index, the number of OTUs per sample, and the pairwise Bray-Curtis dissimilarities were calculated using the R packages vegan v. 2.5-2 (56) and phyloseq v. 1.24.0 (57).

Twelve extraction control samples were also included to assess potential contamination during DNA extraction and sequencing. Any OTUs that were identified as predominant in the negative controls were removed prior to analysis.

### Estimation of *Lactobacillus* abundance from NS using qPCR.

Because of the semiselective nature of MRS plates, accurate counts of *Lactobacillus* spp. could not be achieved. Instead, *Lactobacillus* abundance was estimated via qPCR. A *Lactobacillus* group-specific PCR primer, S-G-Lab-0677-a-A-17, was used to selectively amplify the 16S rRNA gene from the members of the *Lactobacillus* genus (58). Each qPCR mixture contained 1× iQ SYBR green Supermix (Bio-Rad Laboratories, Inc.), 0.4 μM (each) primer, 0.1 μg/μl bovine serum albumin (BSA) (New England Biolabs, Pickering, ON, Canada), and 25 ng of DNA extracted from the NS, in a total volume of 25 μl. A CFX96 Touch real-time PCR detection system (Bio-Rad Laboratories, Inc.) with the following conditions was used: an initial denaturation at 95°C for 3 min, followed by 40 cycles at 95°C for 25 s, 50°C for 30 s, and then 72°C for 45 s. Standard curves (10^2^ to 10^6^ gene copies) were produced using the pDrive cloning vector (Qiagen Inc.) containing the PCR product from a *Lactobacillus* strain (*L. paracasei* 57A). A melt curve analysis was performed following amplification for all qPCRs to ensure that only target genes were amplified.

### Statistical analysis.

The mean *M. haemolytica* counts for each animal were obtained by averaging CFU from the two NS collected per animal (right and left nasal cavities). Bacterial counts, rectal temperatures, and respiratory rates were compared between treatment groups (BT + Mh and Mh). The *M. haemolytica* counts were natural log transformed prior to statistical analysis. All data were analyzed as a randomized complete block design with repeated measurements using a linear mixed-effects model. This model accounted for the repeated measurements and also adjusted for day of sampling and *M. haemolytica* counts before challenge (i.e., baseline). Animal (*n* = 24) was included as a random effect. The parameters were estimated using restricted maximum likelihood. All analyses were done in R version 3.5.0 (R Core Team, Vienna, Austria), and a *P* value of <0.05 was considered significant.

The number of 16S rRNA gene sequences in each NS and TTA was randomly subsampled to 3,850 and 25,500 sequences, respectively, prior to the calculation of alpha-diversity metrics and the Bray-Curtis dissimilarities. Permutational multivariate analysis of variance (PERMANOVA) with 10,000 permutations was used to determine the effect of treatment and sampling time on the microbial community structure. Differentially abundant OTUs in the nasal microbiota of the BT and BT + Mh groups were determined using DESeq2 (59). Only those OTUs found in at least 10% of samples were included in the analysis, and the Benjamini-Hochberg procedure was used to account for multiple comparisons.

The relative abundance of specific taxa from the NS and TTA samples and the qPCR data, as well as serum cytokine concentrations, were analyzed using the GLIMMIX procedure in SAS (SAS 9.4; SAS Institute Inc., Cary, NC). The individual, block, treatment, and time were included in the CLASS statement. The models were “generalized” due to the specification of residual distributions that were not Gaussian normal. Models were “mixed” due to the inclusion of fixed effects (treatment-nested-in-time and time) and random effects (block and individual). Variance heterogeneity was modeled using a “RANDOM_RESIDUAL_/GROUP = Treatment*Time” statement. Residual distributions and covariance structures were selected for each genus based on the model fit statistics, i.e., Bayesian information criterion (BIC). Preliminary models that specified the beta-binomial distribution did not converge. Therefore, alternative distributions were tested: gamma, inverse Gaussian, lognormal, shifted t, Gaussian normal, exponential, and geometric.

Path analysis was performed to evaluate the potential interrelationship structure among the nasal microbial community. Path analysis is a member of the structural equation modeling tools that enable identification of causal relationships between the measured variables (60). For path modeling, data on the 10 most relatively abundant genera in the nasal microbiota were broken into two subsets based on treatment group. Pearson’s product-moment correlation coefficient detects the strength and direction of linear relationships among variables, and the use of Spearman’s correlation coefficients allows for nonlinear (monotonic increasing or decreasing) relationships among the studied variables. Therefore, path analysis was based on the matrix Spearman rank-based correlation coefficients for each subset that were produced using SAS PROC CORR with the SPEARMAN option (SAS 9.4). In the initial model, time and the relative abundance of *Lactobacillus* spp. (the BT inoculation) were exogenous (predictor or independent) variables. In path diagrams, single-headed arrows that represent causal paths point from but never point to exogenous variables. The sources of variability in the exogenous variables are not included in the model. The initial model hypothesized that the experimental variables (time and *Lactobacillus*) predicted variability in the relative abundance of the studied genera (which were therefore hypothesized to be endogenous or criterion variables). As such, the initial model was entered into SAS PROC CALIS:

$$\begin{array}{c} \text{Time. }\mathrm{Lactobacillus}\\ ↓\\ \{\mathrm{Mannheimia},\mathrm{Moraxella},\mathrm{Acinetobacter},\mathrm{Bifidobacterium},\\ \mathrm{Streptococcus},\mathrm{Lactobacillus},\mathrm{Prevotella},\mathrm{Bacteroides},\mathrm{Klebsiella}\} \end{array}$$

Path models were modified by adding and subtracting paths and covariance using the PATH and PCOV statements, respectively, based on suggestions made by Lagrange modifier statistics that were obtained by the inclusion of the MODIFICATION option in the PROC CALIS statement. Time was exogenous, and endogeneity was not considered with respect to time. Lower values of Schwarz’s Bayesian criteria (SBC) indicated superior model fitting, in comparison to the initial hypothetical model, and therefore the plausibility and appropriateness of the respective modified model structures.

### Data availability.

Raw sequence data are available from the NCBI Sequence Read Archive under BioProject accession PRJNA589656.
