# Supplementary figures and images for: Intranasal Bacterial Therapeutics Reduce Colonization by the Respiratory Pathogen Mannheimia haemolytica in Dairy Calves
Source: mSystems. 2020 Mar 3;5(2):e00629-19. doi: 10.1128/mSystems.00629-19 (PMC7055656; doi:10.1128/mSystems.00629-19)

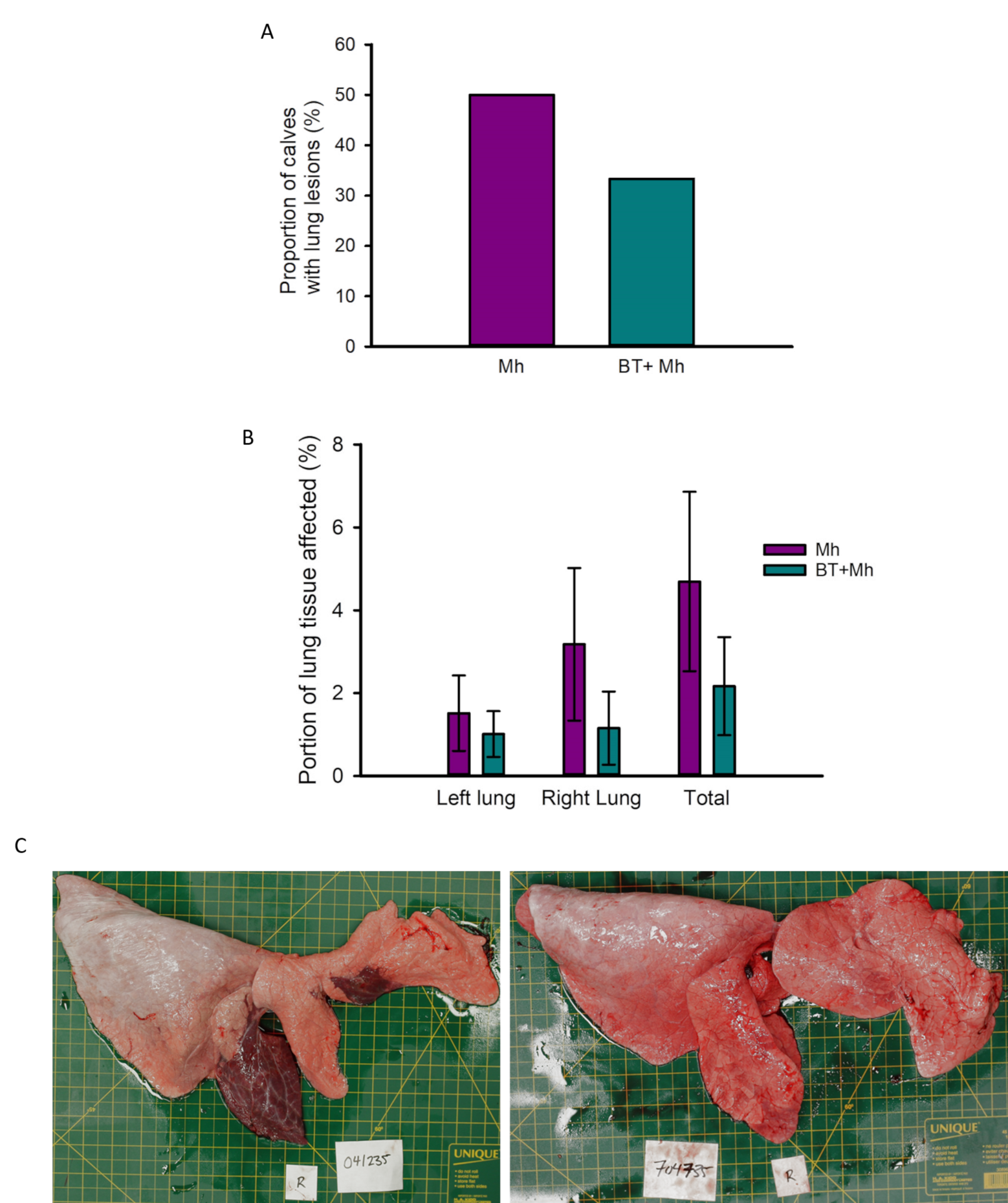

Supplement: FIG S1 [file mSystems.00629-19-sf001.tif]

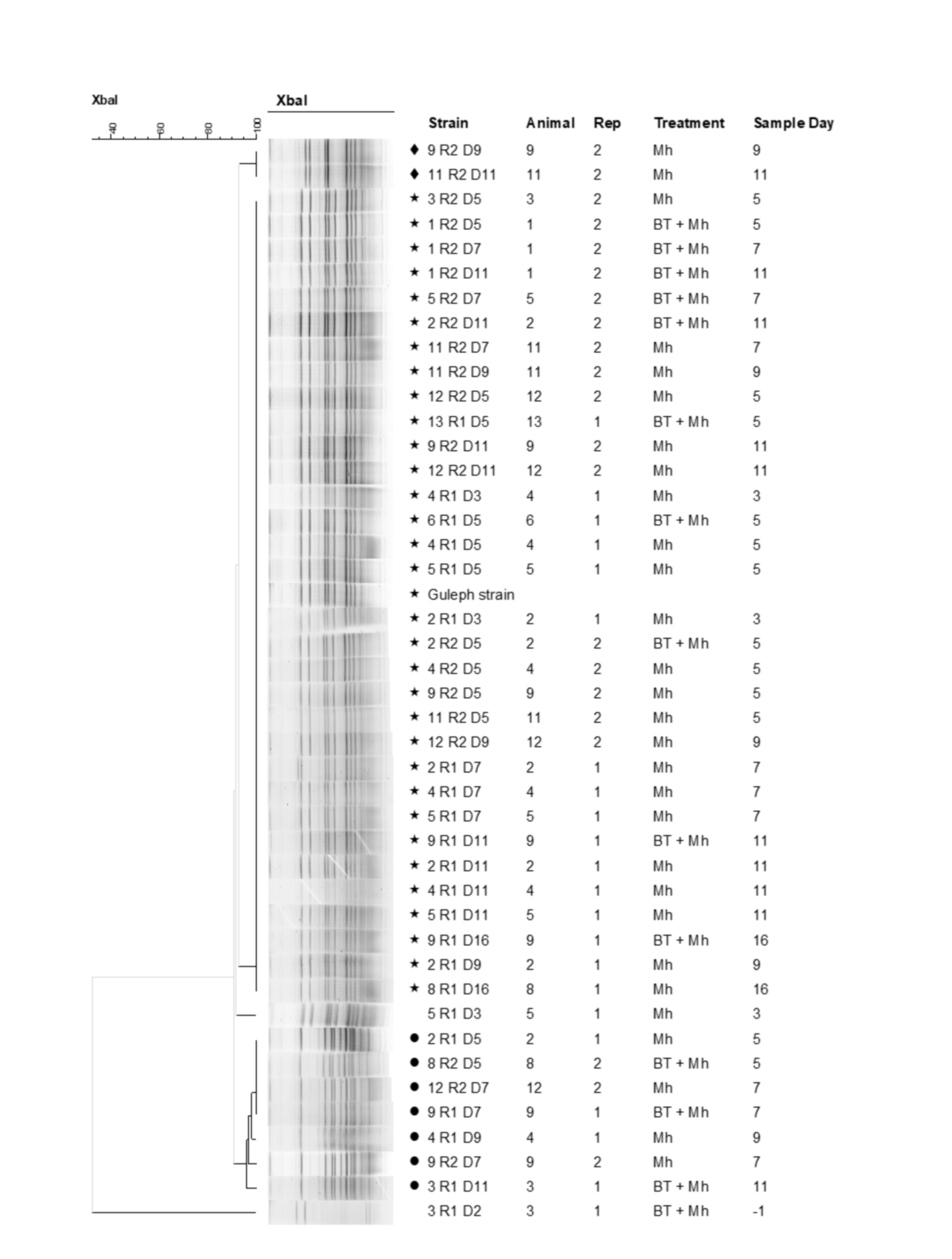

Supplement: FIG S2 [file mSystems.00629-19-sf002.tif]

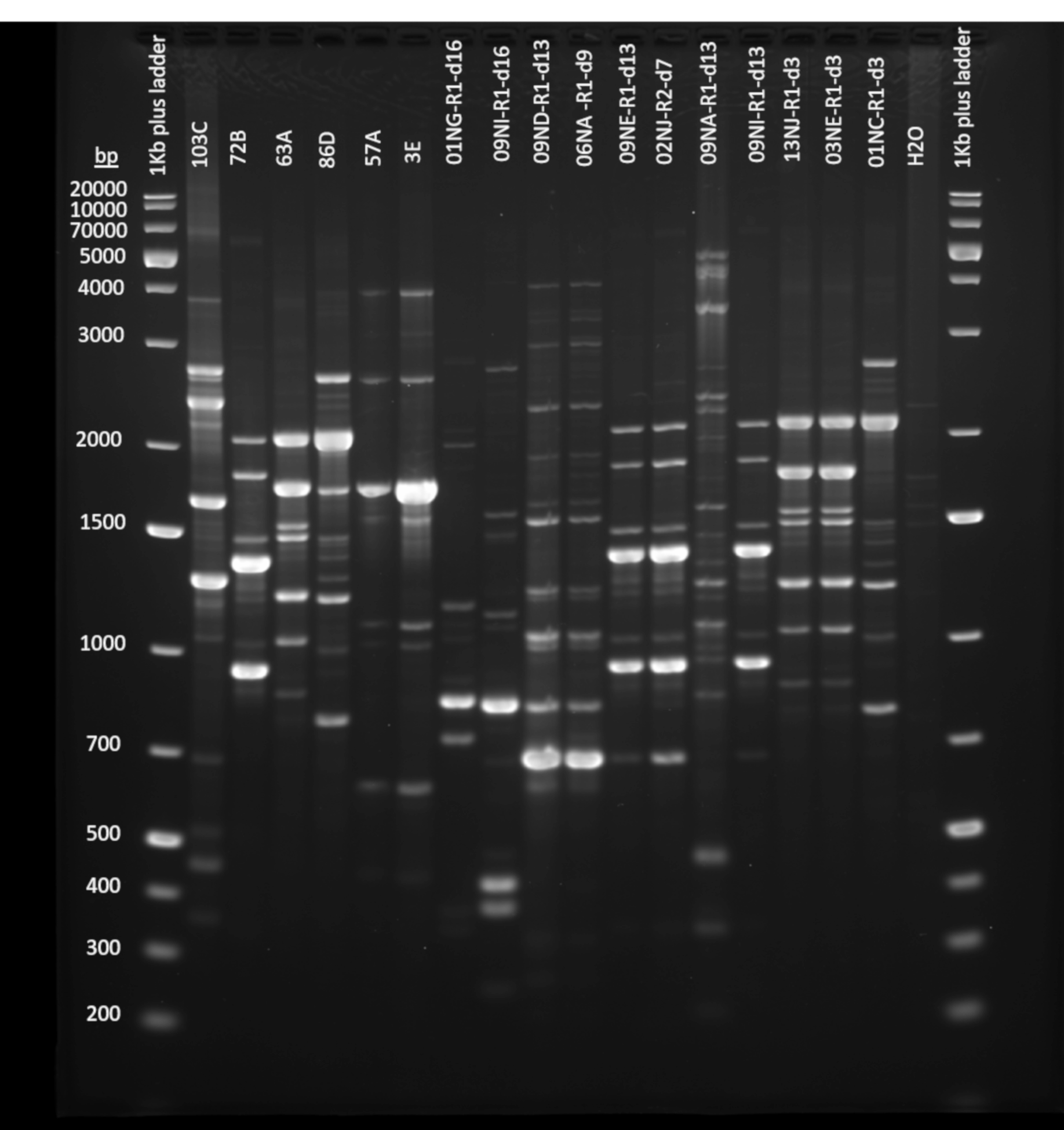

Supplement: FIG S3 [file mSystems.00629-19-sf003.tif]
