# Supplementary material for: Intranasal Bacterial Therapeutics Reduce Colonization by the Respiratory Pathogen Mannheimia haemolytica in Dairy Calves
Source: mSystems. 2020 Mar 3;5(2):e00629-19. doi: 10.1128/mSystems.00629-19 (PMC7055656; doi:10.1128/mSystems.00629-19)
Supplement: TABLE S1 [file mSystems.00629-19-st001.pdf]

Supplementary Table S1.

	Independent variable and level [a]	Estimated mean (SE)	*P*-value
Rectal temperature (ºC)			
	Intercept	5.357 (0.2790)	0.0000
	Mh	Ref.	
	BT + Mh	-0.0403 (0.3947)	0.9196
Respiratory rate (Breaths per minute)			
	Intercept	24.95 (1.367)	0.0000
	Mh	Ref.	
	BT + Mh	0.0476 (0.881)	0.9574
	Day of sampling	-0.2646 (0.122)	0.0320

[a] Random effect = animal (n = 24)
