# Supplementary material for: Intranasal Bacterial Therapeutics Reduce Colonization by the Respiratory Pathogen Mannheimia haemolytica in Dairy Calves
Source: mSystems. 2020 Mar 3;5(2):e00629-19. doi: 10.1128/mSystems.00629-19 (PMC7055656; doi:10.1128/mSystems.00629-19)
Supplement: TABLE S2 [file mSystems.00629-19-st002.pdf]

Supplementary Table S2.

Treatment	Sampling day	72B (*L. amylovorus*)	86D (*L. buchneri*)	63A (*L. buchneri*)	103C (*L. curvatus*)	3E (*L. paracasei*)	57A (*L. paracasei*)
BT + Mh	-1	0	0	0	0	0	0
	3	0	6 (50%)	6 (50%)	0	4 (33%)	4 (33%)
	5	0	0	0	0	1 (8%)	1 (8%)
	7	2 (16%)	2 (16%)	0	0	0	0
	9	0	0	0	0	0	0
	11	0	0	0	0	0	0
	13	2 (16%)	0	0	0	0	0
	16	0	0	0	0	0	0
Mh	-1	0	0	0	0	0	0
	3	0	0	0	0	0	0
	5	0	0	0	0	0	0
	7	0	0	0	0	0	0
	9	0	0	0	0	0	0
	11	0	0	0	0	0	0
	13	0	0	0	0	0	0
	16	0	0	0	0	0	0

[a]Identification of BT strains was completed by two steps: in step one, a *Lactobacillus* genus-specific PCR was performed to identify the *Lactobacillus* isolates; in the second step, the $(GTG)_5$ Rep PCR was performed on those *Lactobacillus* strains.
