# Supplementary material for: Intranasal Bacterial Therapeutics Reduce Colonization by the Respiratory Pathogen Mannheimia haemolytica in Dairy Calves
Source: mSystems. 2020 Mar 3;5(2):e00629-19. doi: 10.1128/mSystems.00629-19 (PMC7055656; doi:10.1128/mSystems.00629-19)
Supplement: TABLE S3 [file mSystems.00629-19-st003.pdf]

Supplementary Table S3.

OTU ID	Mean	log2 (Fold Change)	FDRI	Phylum	Family	Genus
OTU57	74.3[a]	24.6[b]	1.6E-15	Firmicutes	*Lachnospiraceae*	NA
OTU5	66.5	24.4	1.8E-15	Proteobacteria	*Moraxellaceae*	*Moraxella*
OTU128	43.9	23.8	7.4E-15	Firmicutes	*Ruminococcaceae*	*Faecalibacterium*
OTU62	26.6	23.1	3.6E-14	Bacteroidetes	*Prevotellaceae*	*Prevotella*
OTU240	23.9	23.0	4.2E-14	Firmicutes	*Ruminococcaceae*	*Butyricicoccus*
OTU164	16.5	22.5	1.3E-13	Firmicutes	*Lachnospiraceae*	*Blautia*
OTU34	16.4	22.5	1.3E-13	Firmicutes	*Streptococcaceae*	*Lactococcus*
OTU31	24.8	22.2	2.1E-13	Actinobacteria	*Bifidobacteriaceae*	*Bifidobacterium*
OTU12	6.9	21.3	2.2E-12	Actinobacteria	*Bifidobacteriaceae*	*Bifidobacterium*
OTU140	6.9	21.3	2.2E-12	Bacteroidetes	*Bacteroidaceae*	*Bacteroides*
OTU167	3.8	20.4	1.6E-11	Firmicutes	*Lactobacillaceae*	*Lactobacillus*
OTU706	3.7	20.4	1.7E-11	Actinobacteria	*Brevibacteriaceae*	*Brevibacterium*
OTU6	1273.1	8.9	6.2E-03	Actinobacteria	*Microbacteriaceae*	NA
OTU14	13.3	-7.5	3.3E-02	Actinobacteria	*Bifidobacteriaceae*	*Bifidobacterium*
OTU107	6.2	-22.6	1.2E-13	Firmicutes	*Streptococcaceae*	*Lactococcus*
OTU189	9.1	-23.1	3.7E-14	Firmicutes	*Veillonellaceae*	*Veillonella*
OTU88	13.9	-23.5	1.5E-14	Bacteroidetes	*Prevotellaceae*	*Prevotella*
OTU365	27.7	-24.6	1.6E-15	Bacteroidetes	*Bacteroidaceae*	*Bacteroides*
OTU23	57.0	-25.6	2.4E-16	Actinobacteria	*Atopobiaceae*	*Olsenella*

[a]Mean abundance values are the mean abundance for each OTU among all Mh and BT + Mh samples.

[b]Positive fold change values indicate OTUs that were enriched in the Mh group and negative values indicate OTUs enriched in the BT + Mh calves.
