# Supplementary material for: Intranasal Bacterial Therapeutics Reduce Colonization by the Respiratory Pathogen Mannheimia haemolytica in Dairy Calves
Source: mSystems. 2020 Mar 3;5(2):e00629-19. doi: 10.1128/mSystems.00629-19 (PMC7055656; doi:10.1128/mSystems.00629-19)
Supplement: TABLE S4 [file mSystems.00629-19-st004.pdf]

Supplementary Table S4.

	Time	*Mannheimia*	*Moraxella*	*Lactococcus*	*Acinetobacter*	*Bifidobacterium*	*Streptococcus*	*Lactobacillus*	*Prevotella*	*Bacteroides*	*Klebsiella*
Time	1	-0.1583	0.0368	-0.1851	-0.2149	-0.0589	-0.2022	-0.0294	0.2295	0.2828	-0.0733
Mannheimia	-0.1583	1	-0.0978	-0.309	-0.0997	-0.2027	0.0804	-0.1091	0.141	-0.0093	-0.0625
Moraxella	0.0368	-0.0978	1	0.1089	0.3531	0.0624	0.2139	0.0428	0.2024	0.1187	0.3395
Lactococcus	-0.1851	-0.309	0.1089	1	0.4001	0.5465	0.2448	0.3124	-0.1989	0.1383	0.1701
Acinetobacter	-0.2149	-0.0997	0.3531	0.4001	1	0.5516	0.3503	0.4798	0.1439	0.0308	0.5915
Bifidobacterium	-0.0589	-0.2027	0.0624	0.5465	0.5516	1	0.2454	0.6726	0.1638	0.2107	0.2911
Streptococcus	-0.2022	0.0804	0.2139	0.2448	0.3503	0.2454	1	0.2052	0.1862	0.2082	0.2037
Lactobacillus	-0.0294	-0.1091	0.0428	0.3124	0.4798	0.6726	0.2052	1	0.1556	0.119	0.427
Prevotella	0.2295	0.141	0.2024	-0.1989	0.1439	0.1638	0.1862	0.1556	1	0.2417	0.1555
Bacteroides	0.2828	-0.0093	0.1187	0.1383	0.0308	0.2107	0.2082	0.119	0.2417	1	0.1509
Klebsiella	-0.0733	-0.0625	0.3395	0.1701	0.5915	0.2911	0.2037	0.427	0.1555	0.1509	1

[a]Correlation analysis was performed on the relative abundance data obtained from all the nasal swabs (n = 91) collected over the course of study.
