# Supplementary material for: Intranasal Bacterial Therapeutics Reduce Colonization by the Respiratory Pathogen Mannheimia haemolytica in Dairy Calves
Source: mSystems. 2020 Mar 3;5(2):e00629-19. doi: 10.1128/mSystems.00629-19 (PMC7055656; doi:10.1128/mSystems.00629-19)
Supplement: TABLE S5 [file mSystems.00629-19-st005.pdf]

Supplementary Table S5.

	Time	*Mannheimia*	*Moraxella*	*Lactococcus*	*Acinetobacter*	*Bifidobacterium*	*Streptococcus*	*Lactobacillus*	*Prevotella*	*Bacteroides*	*Klebsiella*
Time	1	-0.0108	-0.0267	-0.3009	-0.3347	-0.0345	-0.2279	-0.163	0.2615	0.3979	-0.2904
Mannheimia	-0.0108	1	-0.0203	-0.2106	-0.1004	-0.169	0.148	-0.1554	0.1795	-0.085	0.033
Moraxella	-0.0267	-0.0203	1	0.0077	-0.015	0.0158	0.0597	0.0356	0.0424	0.174	0.0309
Lactococcus	-0.3009	-0.2106	0.0077	1	0.3771	0.2681	0.0933	0.1413	-0.3348	0.1138	0.4044
Acinetobacter	-0.3347	-0.1004	-0.015	0.3771	1	0.6	0.3774	0.5244	0.0918	0.1516	0.639
Bifidobacterium	-0.0345	-0.169	0.0158	0.2681	0.6	1	0.3232	0.5408	0.2765	0.4676	0.3511
Streptococcus	-0.2279	0.148	0.0597	0.0933	0.3774	0.3232	1	0.4223	0.2441	0.1425	0.3163
Lactobacillus	-0.163	-0.1554	0.0356	0.1413	0.5244	0.5408	0.4223	1	0.2022	0.217	0.3205
Prevotella	0.2615	0.1795	0.0424	-0.3348	0.0918	0.2765	0.2441	0.2022	1	0.3599	0.1123
Bacteroides	0.3979	-0.085	0.174	0.1138	0.1516	0.4676	0.1425	0.217	0.3599	1	0.2134
Klebsiella	-0.2904	0.033	0.0309	0.4044	0.639	0.3511	0.3163	0.3205	0.1123	0.2134	1

[a]Correlation analysis was performed on the relative abundance data obtained from all the nasal swabs (n = 88) collected over the course of study.
