# Supplementary material for: Intranasal Bacterial Therapeutics Reduce Colonization by the Respiratory Pathogen Mannheimia haemolytica in Dairy Calves
Source: mSystems. 2020 Mar 3;5(2):e00629-19. doi: 10.1128/mSystems.00629-19 (PMC7055656; doi:10.1128/mSystems.00629-19)
Supplement: TABLE S6 [file mSystems.00629-19-st006.pdf]

Supplementary Table S6.

	Unmodified model: (Time, Lactobacillus) → (Mannheimia, Moraxella, Acinetobacter, Bifidobacterium, Streptococcus, Lactobacillus, Prevotella, Bacteroides, Klebsiella)	Modified model 1 (Mh group): Time → (*Bacteroides, Prevotella, Mannheimia, Acinetobacter*), *Lactobacillus* → *Bifidobacterium*, (*Klebsiella, Bifidobacterium*) → *Acinetobacter, Lactococcus* → *Mannheimia Acinetobacter* → *Streptococcus, Streptococcus* → (*Bacteroides, Prevotella*), (*Bifidobacterium, Prevotella*) → *Lactococcus, Acinetobacter* ↔ *Moraxella* ; 0 = {(*Lactobacillus, Moraxella, Klebsiella*) ↔ Time, *Lactobacillus* ↔ *Moraxella*}	Modified model 2 (BT + Mh group): *Klebsiella* → (*Lactobacillus, Acinetobacter, Bacteroides, Lactococcus*) Time → (*Lactococcus, Klebsiella, Bacteroides, Bifidobacterium*), *Lactobacillus* → (*Bifidobacterium, Streptococcus*), *Bifidobacterium* → *Bacteroides, Acinetobacter* → *Bifidobacterium, Mannheimia* → *Streptococcus, Bacteroides* → *Prevotella, Lactococcus* ↔ *Prevotella, Bacteroides* ↔ *Prevotella, Lactobacillus* ↔ *Acinetobacter*; 0 = {*Mannheimia* ↔ (Time, *Moraxella, Lactobacillus*), *Moraxella* ↔ (Time, *Lactobacillus*), *Lactobacillus* ↔ Time}
Mh	281.1053	154.4841	235.4901
BT + Mh	283.2544	220.6100	163.6963
Model fit statistics (model 1 to Mh group data, model 2 to BT + Mh group data)			
Iterations		7	9
Chi-square		χ^2_{39} = 32.6909, p = 0.7519	χ^2_{38}=38.3309, p = 0.4545
RMSEA		< .0001	< .0001
Bentler-Bonett NFI		0.8863	0.8724
Bentler-Bonnett non-NFI		1.0383	0.9980
Stability coefficient of reciprocal causation		0	0
