# Supplementary material for: Intranasal Bacterial Therapeutics Reduce Colonization by the Respiratory Pathogen Mannheimia haemolytica in Dairy Calves
Source: mSystems. 2020 Mar 3;5(2):e00629-19. doi: 10.1128/mSystems.00629-19 (PMC7055656; doi:10.1128/mSystems.00629-19)
Supplement: TABLE S7 [file mSystems.00629-19-st007.pdf]

Supplementary Table S7.

Relative abundance	$R^2_{Model\ 1}$	$R^2_{Model\ 2}$
Acinetobacter	0.5147	0.4083
Bacteroides	0.1752	0.4494
Bifidobacterium	0.4524	0.4786
Lactococcus	0.4100	0.1944
Mannheimia	0.1621	.
Prevotella	0.1257	0.1171
Streptococcus	0.1190	0.2484
Klebsiella	.	0.0843
Lactobacillus	.	0.1027
